# Role of interventional oncology in hepatocellular carcinoma: Future best practice beyond current guidelines

**DOI:** 10.1259/bjr.20220379

**Published:** 2022-08-04

**Authors:** Piyush Goyal, Riad Salem, Samdeep K. Mouli

**Affiliations:** 1 Department of Radiology, Section of Interventional Radiology, Northwestern Feinberg School of Medicine, Chicago, Illinois, United States

## Abstract

Hepatocellular carcinoma (HCC) is a leading cause of cancer-related deaths globally. Liver transplant remains the goal of curative treatment, but limited supply of organs decreases accessibility and prolongs waiting time to transplantation. Therefore, interventional oncology therapies have been used to treat the majority of HCC patients, including those awaiting transplant. The Barcelona Clinic Liver Cancer (BCLC) classification is the most widely used staging system in management of HCC that helps allocate treatments. Since its inception in 1999, it was updated for the fifth time in November 2021 and for the first time shaped by expert opinions outside the core BCLC group. The most recent version includes additional options for early-stage disease, substratifies intermediate disease into three groups, and lists alternates to Sorafenib that can double the expected survival of advanced-stage disease. The group also proposed a new BCLC staging schema for disease progression, and endorsed treatment stage migration (TSM) directly into the main staging and treatment algorithm. This article reviews the recent developments underlying the current BCLC guidelines and highlights ongoing research, particularly involving radioembolization, that will shape future best practice.

## Introduction to hepatocellular carcinoma

Primary liver cancer is the third leading cause of cancer-related death globally. In 2020, there were estimated 905,677 new cases and 830,180 deaths.^1^ Ninety percent of primary liver cancer can be attributed to hepatocellular carcinoma (HCC), and HCC has greater than 50% recurrence after 5 years despite surgical resection or ablation.^2,3^

The purpose of this article is to provide a review of recent interventional oncology developments and infer their impact on future treatment guidelines for HCC. A brief introduction on current guidelines and treatment options is given before detailing the locoregional toolbox: ablation, chemoembolization, and radioembolization. Finally, speculation on newly emerging research is provided, along with their potential long-term treatment implications for patients suffering with HCC.

## The BCLC guidelines for management of hepatocellular carcinoma

Although multiple staging systems for HCC have been proposed, the most widely used prognostic staging system is the Barcelona Clinic Liver Cancer (BCLC) staging system.^4^ BCLC system uses tumor burden, the extent of underlying liver disease (Child-Pugh, MELD, ALBI) and ECOG Performance Scale (PS) to stage patients from a very early stage (0) through a terminal stage (D) disease. The staging system also suggests surgical and non-surgical first-line treatments. First-line locoregional treatments include: ablation in stages 0 and A patients who are not candidates for surgery, and chemoembolization in stage B patients with well-defined nodules. Extrapolation from the multiregional BRIDGE study suggests that the approximate percentage of patients in North America and Europe at time of diagnosis in BCLC stages 0, A, B, C, and D are 5%, 27%, 11%, 48% and 9%, respectively.^5^

**Figure 1.**
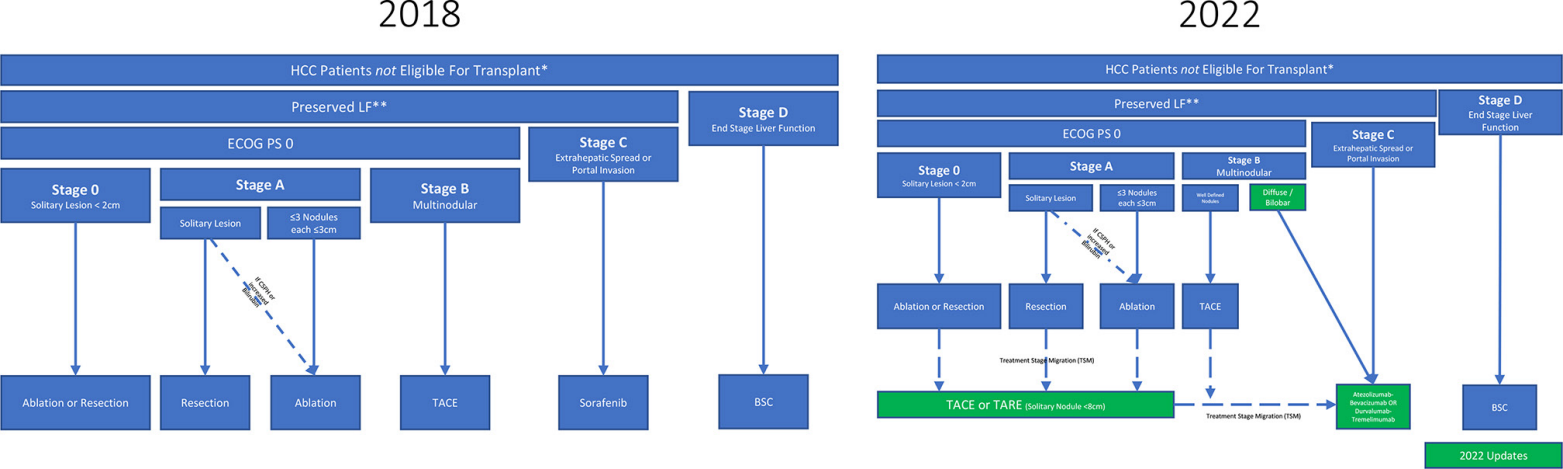
Simplified hepatocellular carcinoma management algorithm in patients not eligible for live transplant. Adopted from the BCLC 2018 (**A**) or BCLC 2022 (**B**) guidelines. Green boxes highlight the recent changes. *Goal should be to bridge-to-transplant or downstage-to-transplant, but relatively few patients are eligible for either **Preserved liver function is a clinical evaluation composed of physical exam findings (lack of tense ascites, jaundice, encephalopathy) and laboratory findings showing *generally* Child-Pugh A and in some cases Child-Pugh B7. However, ALBI, MELD-Na, and AFP scores should also be evaluated, particularly when patient is in consideration for eventual transplant. Abbreviations: HCC = hepatocellular carcinoma LF = liver function TARE = transarterial radioembolization TACE = transarterial chemoembolization BSC = best supportive care PS = performance score CSPH = clinically significant portal hypertension

Figure 1 depicts a summarized version of the most recent (2022) BCLC staging system, alongside the previous (2018) version for comparison. This update was the first time expert opinions outside the BCLC core group were considered, and the concept of treatment stage migration (TSM) was formalized into the algorithm.^6^ TSM relies on multidisciplinary evaluation of patients’ characteristics and local expertise to sometimes recommend a more advanced stage therapy than the first option suggested by BCLC staging.

Notable changes in the most recent iteration of the BCLC guidelines include: 1) inclusion of chemoembolization and radioembolization for early stages of disease where ablation or surgery have either failed or are not feasible, 2) substratification of intermediate stage B group into three populations with subsequent treatment recommendations, and 3) in advanced disease, stage C replacement of tyrosine kinase inhibitor Sorafenib as first line with either a (Programmed death-ligand 1) PDL1-VEGF (Vascular endothelial growth factor) inhibitor combination (Atezolizumab-Bevacizumab) or dual PDL1-CTLA4 (cytotoxic T-lymphocyte-associated protein 4) checkpoint inhibitors (Durvalumab-Tremelimumab). Results of the LEGACY (Local radioEmbolization using Glass Microspheres for the Assessment of Tumor Control with Y-90), IMBrave150 (Atezolizumab plus Bevacizumab in Unresectable Hepatocellular Carcinoma), and HIMALAYA (Study of Durvalumab and Tremelimumab as First-line Treatment in Patients With Advanced Hepatocellular Carcinoma) trials shaped these changes and are discussed in sections ahead.

Radiologic studies are a key component of initial staging and post-treatment monitoring. After much evolution, the two main imaging response criteria currently used to evaluate treatment response and progression are RECIST (tumor size-based) and modified RECIST (mRECIST, tumor arterial-enhancement based).^7,8^ The recent BCLC guidelines utilize RECIST to evaluate patients with radiologic progression. Indeed, the 2022 BCLC guidelines also include a second flowchart offering stratification of patients who demonstrate radiologic progression after initial treatment. HCC progression has heterogenous implications – recurrence or growth at known sites has better prognosis and may be amenable to repeat treatment with the same modality previously used; while *de novo* extrahepatic involvement may warrant a new aggressive treatment course, such as systemic therapy.

## Overview of treatment options

Initial management of HCC involves evaluating whether the patient is eligible to receive a liver transplant or liver resection. However, a small percentage of patients are eligible for either.^9^ The criteria for surgical intervention are nuanced and guide multidisciplinary management.^10^ Briefly, patients with HCC who, after a 6-month waiting period, fit within the Milan criteria (one lesion with a diameter less than or equal to 5 cm or ≤3 lesions each with a diameter less than or equal to 3 cm with no macrovascular or extrahepatic invasion) are awarded exception points to gain priority on the waiting list.^11^ However, many have advocated for expanding the Milan criteria to be less restrictive, such as the UCSF criteria or the up to seven criteria.^9^ Regarding surgical resection, it is best offered to very early- or early-stage patients with a solitary lesion without vascular invasion. Up to three-fourths of a *healthy* liver can be resected with no impact on patient survival. Of course, a much smaller portion, if any, can be resected of a cirrhotic liver.^12^ Surgical candidates must usually have a hepatic venous pressure gradient <10 mm Hg and adequate post-surgical remnant liver to ensure that liver function does not deteriorate beyond the pre-operative level. Additional surgical considerations for HCC have been discussed elsewhere.^13^

Getting HCC patients eligible for liver transplant remains an emphasis of the BCLC guidelines. This includes IR interventions to “bridge to transplant” or to “downstage to transplant”, the latter being formally drawn into the BCLC 2022 algorithm.^14^ Approximately 10% of HCC patients on a transplant waitlist drop off due to tumor progression that puts them outside local eligibility criteria.^14^ Bridging to transplant involves using locoregional therapies to maintain or lower tumor burden until successful transplant. In contrast, downstaging refers to use of locoregional therapy in patients outside transplant criteria, to decrease their tumor burden to within the Milan criteria, such that the patient would be eligible for a liver transplant.^15^ Finally, in patients undergoing partial hepatectomy, a pre-operative portal vein embolization of the supply to the resected segment can induce hypertrophy in the spared segments.^16^ In fact, a recent study has demonstrated that transarterial radioembolization can achieve similar results with improved tumor control and prevent tumor progression during the hypertrophy period.^17^ Additionally, the longer time it takes to achieve hypertrophy with radioembolization compared to bland embolization can actually be used to identify subpopulations with aggressive disease that may benefit from a different treatment approach.^18^

Although not the focus of this review, it is important to mention two advancements in systemic therapy in the last 2 years that have doubled the median life expectancy of patients with advanced disease BCLC Stage C. HIMALAYA and IMbrave150 were both multicenter open-label Phase III trials that randomized unresectable HCC patients to either an immune checkpoint inhibitor regimen or Sorafenib. In both trials, overall survival and adverse event rate of patients on Sorafenib was worse. The results from the IMbrave150 trial and HIMALAYA trial have replaced Sorafenib as the first-line treatment for advanced HCC with the combination of anti-PDL1 Atezolizumab and Bevacizumab or anti-CTLA4 Tremelimumab and anti-PDL1 Durvalumab, respectively.^19,20^ Note that the molecular underpinnings and evolution of systemic therapy have been reviewed elsewhere.^21^ However, combination of systemic agents with locoregional therapies will be discussed below.

The goal for cancer treatment is to remove the tumor(s) and minimize recurrence. However, most HCC patients are not surgical candidates due to comorbidities, unresectable disease, or metastatic disease. This is where the majority of interventional oncology efforts detailed below are directed.

## The interventional oncology toolbox: Ongoing developments and future best practice

### Percutaneous ablation

Resection and ablation are curative techniques limited to very early- and early-stage disease, with an expected median OS >5 years for either stage. But fewer than 30% of HCC patients are considered resectable.^22^ Both radiofrequency ablation (RFA) and microwave ablation (MWA) are minimally invasive techniques to thermally ablate and induce coagulative necrosis in small lesions, generally <3–5 cm.^23^ Since MWA is a newer technology, more data with RFA are currently available.^24^

In patients amenable to surgical resection, metanalyses have shown comparable overall survival (OS) between surgery and RFA.^25^ Given equivocal results, institutional experience and patient preferences should guide treatment decisions in early-stage disease.^26^ Currently for RFA in stage 0 and A diseases, complete response (CR) based on imaging is >90% and 10-year OS is approximately 30%.^27^ A key limitation to thermal ablation includes risk of incomplete necrosis, usually in larger lesions that likely underly recurrence. This is partly due to RFA’s susceptibility to the “heat-sink” effect through adjacent vessels which limit the ablation zone.^28^ MWA is not limited by this, and current evidence suggests equivalent tumor control when compared to RFA.^29,30^ Hence, MWA has largely replaced RFA in current clinical practice for liver ablation. Still, limitations to percutaneous ablation include risk of seeding, some inaccessible tumor locations, and biliary complications if performed in proximity to central bile ducts.^31^

Patients often receive locoregional therapy prior to liver transplant. A retrospective study using the European Liver Transplant Registry showed RFA to have the highest 5-year OS of 80.9% post-liver transplant compared to 67.6% with chemoembolization and 65.8% with no bridging or downstaging treatment.^32^ However, chemoembolization remains the most common bridging and downstaging treatment.^33^ Still, the transplant drop-out rates between the two modalities are currently comparable. It remains to be seen if drop-out rates with ablation may be further improved as more long-term data with MWA become available.^33^

The combination of ablation with immunotherapy is being explored for treatment of stage B and C diseases. Preclinical studies have postulated that inflammation induced by RFA may cause cancer progression via immunosuppression, while others demonstrate that ablation enhances host adaptive immunity.^34,35^ Although no combination of percutaneous ablation and immunotherapy has been currently endorsed, a small feasibility study in advanced disease patients who failed Sorafenib therapy, tested anti-CTLA4 Tremelimumab with RFA and cryoablation.^36^ With a median follow-up of about 3 years, the overall medial survival was 9.2 months for RFA and 15 months for cryoablation. Both combinatorial and adjuvant therapy trials with different molecular targets are currently underway: PDL1 (NCT03847428) and PD1 (NCT03337841, NCT03383458, NCT03753659). Further trials based on the results from these early studies may possibly define a role for ablation in intermediate and advanced disease. In summary, ablation remains a very important treatment option both as a curative treatment, and in bridge-to-transplant and downstaging-to-transplant situations.^37^

## Intra-arterial therapies

### Bland embolization

The goal of hepatic artery bland embolization is occlusion of the arterial supply of a tumor and induction of ischemic necrosis. However, its role has become more limited with the adoption of more advanced embolotherapies such as chemoembolization or radioembolization. Reflecting this change, neither the EASL nor the AASLD practice guidelines, recommends bland embolization as a locoregional therapy. Indeed in 2010, a study by Malagari et al demonstrated that DEB-TACE had superior response, lower recurrence, and longer time to progression compared to bland embolization.^38^ There was no difference however in survival at one year.

On the other hand, a recent randomized Phase II study challenged the additive benefit of Doxorubicin in arterial embolization; suggesting that the majority of treatment benefit arose from selective arterial embolization only.^39^ 101 patients with BCLC stage A through C were randomized to bland embolization or DEB-TACE. There was no significant difference reported in OS or PFS. At 12 months, the mRECIST response rate was also equivalent. There is some debate as to whether the optimal patient population was selected for the study, as patients with portal invasion and extrahepatic disease were included.^40^ Nonetheless, bland hepatic arterial embolization may still have a limited role in patients for whom drug effects from chemoembolization are intolerable, or lesions with specific collateralized extrahepatic vascular supply.^41^ Assessment of vascular variations, particularly of the hepatic arteries, is critical to minimizing unintended off target effects of transarterial therapies.^42^

### Trans-arterial chemoembolization

Modern transarterial chemoembolization (TACE) has evolved from two landmark studies that demonstrated improved survival compared to conservative management of unresectable disease, albeit in patients with Child-Pugh (CP) A liver function.^43,44^ Technical considerations on both conventional TACE (cTACE) and drug-eluting beads TACE (DEB-TACE) have been previously described.^45,46^ Briefly, the mechanism of TACE involves the combination of ischemic necrosis caused by bland embolization with the vascular occlusion also preventing “washout” of the chemotherapy. The chemotherapeutic is usually Doxorubicin loaded spheres in DEB-TACE, but can be a combination of Doxorubicin, Cisplatin or mitomycin C emulsified in lipiodol in cTACE. Hence, the proposed advantage of TACE is in maximizing its tumoral cytotoxicity and minimizing systemic toxicity. For reference, the size of particles used in cTACE or DEB-TACE ranges from 100to 500μm.^47^ And gelfoam is the most used occlusive particle in cTACE, whereas simultaneous embolization and occlusion is achieved with DEB-TACE.

While DEB-TACE was developed with the aim of standardizing local delivery of chemotherapy, both cTACE and DEB-TACE are currently used based on institutional expertise, as definitive prospective randomized control trials are pending.^48^ The main contraindication to TACE remains portal venous thrombosis.

Indeed, as per the current AASLD, EASL, and APASL practice guidelines, TACE is the recommended initial treatment for intermediate HCC patients not eligible for a transplant, with an expected median survival of >2.5 years after treatment.^10,49^ However, in the past decade, much innovation around TACE has been studying its applicability in conjunction with systemic agents; and the results to date have been mixed.^3^ The 2016 global SPACE trial randomized intermediate stage multinodular HCC patients to DEB-TACE with kinase inhibitor Sorafenib (*n* = 154) or DEB-TACE plus placebo (*n* = 153). Median follow-up was 9 months. No significant difference in median TTP (5.6vs 5.5 months) or OS (HR 0.61–1.33) was noted.^50^ However, authors noted possibly better Sorafenib tolerance in Asian patients. This hypothesis is strengthened by the positive results of the recent TACTICS trial in Japan that randomized unresectable HCC patients to cTACE plus Sorafenib or cTACE alone. Compared to SPACE, Sorafenib was administered at a lower dose for longer duration.^51^ In TACTICS, the authors reported significant PFS improvement of 26.7 vs 16.4 months. Thus, with the recent BCLC Stage B changes, further substratification of intermediate stage patients may yield cohorts that respond ideally to varying combinations of TACE and immunotherapy.^52^ And alongside the discussion of whether Sorafenib with TACE has any meaningful long-term benefit in BCLC B patients, there need to be studies to determine how best to sequence locoregional therapy with systemic agents.

Immune checkpoint inhibitors may also play a role in combination with embolotherapies. Locoregional treatment of tumors may induce release of novel antigens that activate the adaptive immune system.^53^ Multiple trials are underway with various PD1, PDL1, and CTLA4 inhibitors in conjunction with TACE, such as EMERALD-1 (PDL1, NCT03778957), LEAP-012 (PD1, NCT04246177), and CheckMate 74W (CTLA4, NCT04340193). Further, the ABC-HCC (PDL1, NCT04803994) and RENOTACE (PD1, NCT04777851) trials will compare TACE head-to-head against checkpoint inhibitors in intermediate-stage patients.

When comparing to other locoregional modalities, a key limitation of TACE remains portal vein occlusion, which can occur in up to half of all HCC patients.^54^ A recent metanalyses on studies up to 2017 suggested that DEB-TACE provided improved overall survival compared to cTACE and TARE at 2 years, although with limited regression on BCLC staging. With regard to safety, TARE had the lowest complication rate, and with the exception of fatigue, DEB-TACE had a better side-effect profile compared to cTACE. However, OR, DCR, and PFS were similar between DEB-TACE and cTACE.^55^ Comparison to radioembolization contrasts with the very recent results of the TRACE trial and is discussed in the next section.^56,57^ Of note, the results of this prospective randomized control trial were released after the BCLC 2022 update, and thus its implications on treatment of intermediate-stage patients will likely shape decision-making in the coming societal practice guidelines.

Advantages of TACE compared to TARE include the radiopacity of lipiodol and the idea that transient non-target embolization will not have lasting effects, unlike TARE where minimal nontarget can cause permanent damage, particularly to the gastrointestinal system. TACE has also been used long before TARE for treatment of HCC, and thus more long-term data are available.^58,59^ TACE can also be performed in patients with high hepatopulmonary shunting, wherein TARE runs the risk of causing radiation-induced pneumonitis. While TARE remains an option for patients not amenable to TACE, an area of research that has yet to be explored is an intentional treatment with combined or sequenced TACE and TARE.^60^

### Trans-arterial radioembolization

Trans-arterial radioembolization (TARE, also known as SIRT - selective internal radiation therapy) involves delivering 20–60 uM-sized yttrium-90 (Y90) microspheres to the tumor arterial supply. Y90 is a pure beta-emitter with 90% of its energy being absorbed within 5 mm, thus limiting radiation to surrounding tissues.^61^ Additionally, Y90 can be imaged via PET and SPECT to establish post-radioembolization localization and dosimetry.^62,63^ Technical considerations for the procedure using both the glass-based TheraSphere (Boston Scientific, Marlborough, MA) and the resin-based SIR-Sphere (Sirtex, Woburn, MA) Y90 microspheres have been previously published.^64^

TARE was initially performed in a “sequential lobar fashion” for palliative purposes in advanced cases with or without portal vein tumor thrombosis (PVTT). However, advancements in techniques and dosimetry have enabled TARE to be adapted to multiple clinical scenarios across the HCC disease spectrum. This includes “radiation lobectomy” with intention of inducing hypertrophy of the untreated lobe (also known as future liver remnant after resection), and “radiation segmentectomy” at higher doses for ablating involved segments, and as a hybrid approach.^65^ As will be detailed below, these advancements have enabled TARE to be utilized across the BCLC stages.^66^

Compared to ischemic and cytotoxic mechanisms of cTACE and DEB-TACE, TARE causes tumor necrosis via radiation-induced DNA damage and eventual cell death. TARE can also be employed in instances of PVTT.^67,68^ A recent propensity-weighted analysis using the US National Cancer Database found TARE to have better outcomes compared to systemic therapy, at least for HCC patients with macrovascular invasion.^69^

The 2016 PREMIERE trial showed Y-90 radioembolization significantly prolonged time to tumor progression (TTP) in unresectable and unablatable patients compared to cTACE.^70^ Patients randomized to TARE (*n* = 24) had a median TTP per WHO/EASL of >26 months versus a median TTP of 6.8 months in patients randomized to cTACE (*n* = 21). In addition, preliminary results from ongoing studies indicate that TARE, at minimum, may provide improved outcomes with improved quality of life in patients with early and intermediate unresectable HCC.^55,71–74^ Most recently the TRACE trial randomized unresectable early-stage and intermediate-stage patients to DEB-TACE (*n* = 34) or TARE (*n* = 38). The trial allowed for patients with ECOG PS one and Child-Pugh B7 to be included. TTP per mRECIST was 17.1 months with TARE vs 9.5 months with DEB-TACE. Further, OS was 30.2 months with TARE compared to 15.6 months with DEB-TACE. In this study, 14 total patients were downstaged and received transplants but median OS of TARE patients remained significantly higher at 27.6 vs 15.6 months, respectively, when censoring for transplant events. Both treatment arms had equivalent tumor burden, ORR, rate of grade ≥3 adverse events, and 30-day mortality. Given the significant benefit observed in the TARE cohort compared to DEB-TACE with a progression HR of 0.36, the study was halted at interim analysis. Both the PREMIERE trial and the TRACE trial used standard dosimetry intending for an absorbed dose of 120 Gy to the perfused lobe.

For the first time, BCLC staging system includes radioembolization as a treatment option, albeit limited to cases involving a single lesion. This is due to the multicenter retrospective LEGACY study that evaluated TARE in 162 patients with an unresectable solitary tumor <8 cm.^75^ Patients received a median dose of 410 Gy. 60.5% of patients were BCLC stage A, while the rest were BLCL stage C due to ECOG PS 1. TARE served as the primary treatment for 128 patients. Post-treatment images were evaluated in a blinded, independent, central review fashion. The confirmed objective response rate per mRECIST was 72.2% and median duration of response was 11.8 months. There was no local progression at 24 months. At three years, the OS was 86.6%. There were three Grade 3 bilirubin and one Grade 3 albumin toxicities reported. However, no adverse events resulted in modification of therapy. The long-term survival and safety data of this early HCC patient cohort will continue to impact treatment decisions in the left half of the BCLC algorithm.^76^

Since TARE (for solitary lesions meeting the LEGACY criteria) was only recently included in the BCLC algorithm for early-stage disease, updated AASLD and EASL guidelines are pending. Additionally, given the inclusion of TSM in the recent BCLC guidelines, it is worth discussing a study published just prior to the update that evaluated overall survival when multidisciplinary tumor board recommendations deviated from BCLC initial recommendations.^77^ In 321 patients with HCC, all BCLC stages were included, and first-line treatment recommendations included resection, transplant, locoregional therapies, systemic sorafenib, and supportive care. In 76% of cases, the tumor board decision deviated from BCLC 2018 recommendations, with the highest discordance in advanced-stage HCC, wherein the majority of patients were treated with TACE (27%) and TARE (59%). This resulted in an OS of 25.4 months vs an expected 10 months with Sorafenib, which is still competitive with today’s expected survival with the newer systemic agents. Still, median OS matched or exceeded expectations in all BCLC stages. A key component of this treatment paradigm was the use of TARE to bridge and downstage advanced stage and terminal stage patients to transplant.

Critical to maximizing effectiveness of TARE will be ensuring that optimal radiation dose is delivered to HCC tumor(s), while minimizing any radiation induced injury to normal tissues including any normal liver parenchyma. The DOSISPHERE Phase II trial sought to test that hypothesis.^78^ Garin et al compared index lesion objective response rate and all lesions’ overall response rate of intrahepatic advanced HCC patients with at least one lesion >7 cm, that were treated with Y90 using a personalized dosimetry-partition model approach (*n* = 31) vs a standard dosimetry-single compartment approach (*n* = 29). In the personalized dosimetry cohort >205 Gy was delivered to the index (largest) lesion, while in the standardized dosimetry cohort, 100–140 Gy was delivered to the involved lobe. The median OS was 26.6 months and 10.7 months, respectively. For those with portal vein thrombosis, the median OS was reported to be 22.9 months and 9.5 months, respectively. Additionally, at 3 months, the ORR per EASL criteria were measured to be 71% in the personalized dosimetry cohort vs only 36% in the standard dosimetry cohort. Furthermore, the overall response rate was 50 and 14%, respectively. Grade 3 or greater adverse events were reported in 60% of patients in the personalized dosimetry cohort vs 76% of patients in the standard cohort. Of importance, DOSISPHERE was intended to enroll up to 254 patients; however, given that the efficacy results of personalized dosimetry exceeded prespecified ORR threshold of 15% at the first interim analysis, the trial was stopped early. The DOSISPHERE results challenge the outcomes of SARAH and SIRveNIB, which have hampered inclusion of TARE for treatment of intermediate disease.^79^ This suggests high-impact future studies will be those that report on patient survival when personalized dosimetry is used, and looking even further ahead, likely in combination with personalized immunotherapy. Additional dosimetric efforts, including the DOORway90 (Duration Of Objective Response with arterial Ytrrium-90) trial evaluating resin-based microspheres with personalized dosimetry, are underway.^80,81^

Additionally, determinants of the clinically safe maximal dose to the liver that can be delivered based on individual patients’ reduced hepatic reserve needs to be further studied. The TARGET (The TheraSphere^™^ Advanced Dosimetry Retrospective Global Study Evaluation in Hepatocellular Carcinoma Treatment) study was a multicenter study that evaluated relationship between tumor absorbed dose, normal liver absorbed dose, and adverse events. Multicompartment dosimetry was performed retrospectively, which found a median tumor absorbed dose of 217.1 Gy and a normal tissue absorbed dose of 48.1 Gy. Results suggest no relationship between Grade three or higher hyperbilirubinemia and normal tissue absorbed dose that ranged from 5.4to 166 Gy.^82^ Although cases of disease progression were excluded from this analysis.

Looking further into the future, randomized-controlled trials evaluating TARE in combination with systemic immunotherapeutics will change the treatment landscape. As detailed above, TACE is undergoing a similar transition. In 2020, Teyateeti et al reported on, among additional cohorts, survival in patients with <50% intrahepatic tumor burden with and without advanced disease features such as macrovascular invasion.^83^ The former cohort was treated with TARE (median dose of 110 Gy) alone, while the later was treated with TARE (median dose of 110 Gy) and the antiangiogenic Sorafenib. The survival was equivalent in both at 21.6 months.

Nonetheless, the newer systemic agents for HCC are also being investigated in combination with TARE. Although a synergistic mechanism is yet to be elucidated, preclinical studies have shown that radiation induces immunomodulatory effects that are amenable to immunotherapy, at least immune checkpoint inhibitors (ICI).^84^ A recent Phase II study in Asia evaluated the RECIST objective response rate in 36 advanced disease patients treated with resin-Y90 followed by the PD-1 inhibitor nivolumab. ORR was determined to be 30.6%.^85^ While finalized results from trials studying combined TACE plus immune checkpoint inhibitor treatment are pending, the ROWAN trial is already underway. This study will evaluate potential safety and synergy of TARE and checkpoint inhibitors Durvalumab and Tremelimumab in Child-Pugh A patients with preserved ECOG PS that are not eligible for curative treatment (NCT05063565). Of note, immunotherapies are not without significant systemic side-effects, including hyperbilirubinemia. In fact, indication of Nivolumab for HCC post-sorafenib treatment was withdrawn after results of CheckMate-459 (Nivolumab versus sorafenib in advanced HCC) were released.^86^

As additional centers gain interventional oncology specialty care, it will be interesting to see how the next iteration of AASLD and EASL management guidelines include TARE. With further standardization of recent advancements in personalized dosimetry and forthcoming data on combination with systemic immunotherapy agents, TARE has the potential to upgrade from being a neoadjuvant to a first-line treatment in patients ineligible for liver transplant. This was recently proposed in a treatment algorithm stratifying patients based on Child-Pugh class as the first decision variable.^87^

## Looking ahead on future practice

HCC is a multifactorial disease, with varying patient preferences and background liver function and varying local expertise and organ supply; and thus, treatment must be adopted with all three concepts in mind. This is partially captured with inclusion of TSM directly into the BCLC algorithm. However, interventional oncology allows for multidirectional treatment migration that requires personalized decision-making for both initial treatments and for subsequent treatments, when necessary.

While a liver transplant remains the gold standard therapy for HCC patients, there is a massive organ shortage. Additionally, almost half of HCC patients present with stage C disease at time of diagnosis and thus do not qualify for a transplant even within the extended criteria. Therefore, combination of locoregional therapies and systemic agents, at varying timepoints in the clinical course, remain the mainstay of treatment for the majority HCC patients. Pending resolution of technical details related to optimal sequencing of each treatment, combination of immune checkpoint inhibitors with TACE and TARE will continue to be explored in patients with intermediate and advanced disease. Chemical modifications to TACE may also be forthcoming. Doxorubicin is the most common chemotherapeutic used in TACE. However, interesting pre-clinical research into novel lipophilic molecules like idarubicin and modulating the tumor microenvironment pH may eventually add new tools to the interventional radiologist’s arsenal.^88,89^

Short hospital stays are desirable for both patients and health systems. It is plausible that continued improvements in percutaneous ablation could replace surgical resection for early-stage diseases. Combination of transarterial chemoembolization with percutaneous ablation is also being studied in both solitary and multinodular disease. The rationale being that the embolic nature of TACE can reduce the heat-sink effect allowing for larger ablation areas, and these larger ablation zones in turn will minimize repeat TACE treatments.^90^ A retrospective study evaluating patients with a single lesion between 3.1 and 5 cm reported a 10-year OS of 41.8% when treated with combined cTACE and RFA vs 28.4 and 11.9% when treated with chemoembolization only or RFA only, respectively.^91^ Further, metanalysis of mostly retrospective studies comparing TACE plus RFA to surgical resection demonstrate equivalent overall survival with shorter hospital stay and lower adverse event rates when undergoing minimally invasive treatment.^92^

More recently, a retrospective study of patients with unresectable HCC stated that those treated with TACE followed by MWA had an OS and PFS of 49.6 months and 12.6 months, respectively, compared to an OS and PFS of 23.3 months and 8.2 months, respectively, in TACE only patients.^93^ The downstaging to transplant rate was higher in the TACE plus MWA group as well. However, given that the patients were not strictly BCLC stage B, additional forthcoming studies with MWA will prove insightful in the patient population that can benefit most from this dual therapy.

Radioembolization may be the best subsequent option available for patients who have recurrence or progression from other modalities. However, given the adaptability of TARE with personalized dosimetry, and possibly voxel-based dosimetry, it is foreseeable that TARE may supersede TACE when local expertise is present. Dosimetry has been personalized from the body surface area method and the single compartment method to the partition model.^94^ However, due to vascularity amongst other reasons, HCC lesions can be heterogenous, both within a single tumor and amongst neighboring tumors. Voxels are even smaller “partitions” that can be used to capture this heterogeneity, and 3D dosimetry-based radiobiologic models could then help determine not only the ideal activity of but also the ideal number of, particles to infuse based on distribution.^95^

Advancements in radioembolization are not without limitations. Given the microembolic nature of radiospheres, consideration to non-target embolization, for example due to reflux or shunting, must be undertaken. For this, a pretreatment ^99m^Tc macroaggregated albumin (99mTc-MAA) angiography study with, now, SPECT/CT scanning is done to evaluate extent of shunting, particularly to the lung and other GI organs. Some vessels can be coiled off prior to treatment to facilitate containment of the microsphere distribution to the intended targets. And for selected early-stage HCC patients, this procedure can be skipped altogether.^96^ Other technical issues such as catheter positioning based on anatomy, impact of arterial spams during MAA scans, and speed of injections remain to be standardized across centers.^97^

Results of combination trials of locoregional therapies with immunotherapy are eagerly awaited. However, timing of systemic therapy with local therapy is varied, immune checkpoint inhibitor-related adverse events (irAEs) are vast, and there is relatively sparse research thus far in predicting side-effect profile based on patient characteristics.^98^ One interesting concept is potentially utilizing immuno-positron emission tomography (immunoPET) to survey the immune response whereby treatment timing could be optimized for maximal therapeutic-effect and minimal sideeffect.^99^ For example, patients in future combination trials could receive a low-dose radiotracer-mAb conjugate just before and after locoregional therapy to visualize antigen expression. Based on these data, patients could be stratified into a variety of dose-timing schema to minimize irAEs and maximize potential abscopal effects.

Recurrence and progression after treatment remains a limitation in TACE and TARE. Thus, the newly provided BCLC classification of radiologic progression is a welcome tool for future research. As the debate between the two-dimensional mRECIST and RECIST criteria continues, computational advances are slowly ushering in functional and three-dimensional-based evaluations that promise to deliver reproducible predictors of early response.^100^ This could perhaps be best utilized to identify treatable BCLC D patients that fall outside liver transplant eligibility, as currently no viable treatment options exist for this group.

Lastly, proportion of HCC due to viral hepatitis is declining with improved vaccination programs, antiviral therapies, and screening. Cirrhosis secondary to metabolic disorders, alcohol and non-alcoholic steatohepatitis (NASH) may soon become the more common risk factors for HCC.^101^ As the proportion of HCC etiology due to NASH increases, the prognostic staging paradigm itself may need to be overhauled to be more precision-medicine based and initially stratified on molecular markers, as opposed to imaging findings.^102–106^ On the treatment side, it is worthwhile to keep an eye on developments in oncolytic viral therapy, mRNA-based vaccines, and micro-RNA cancer screening.^107,108^

**Figure 2.**
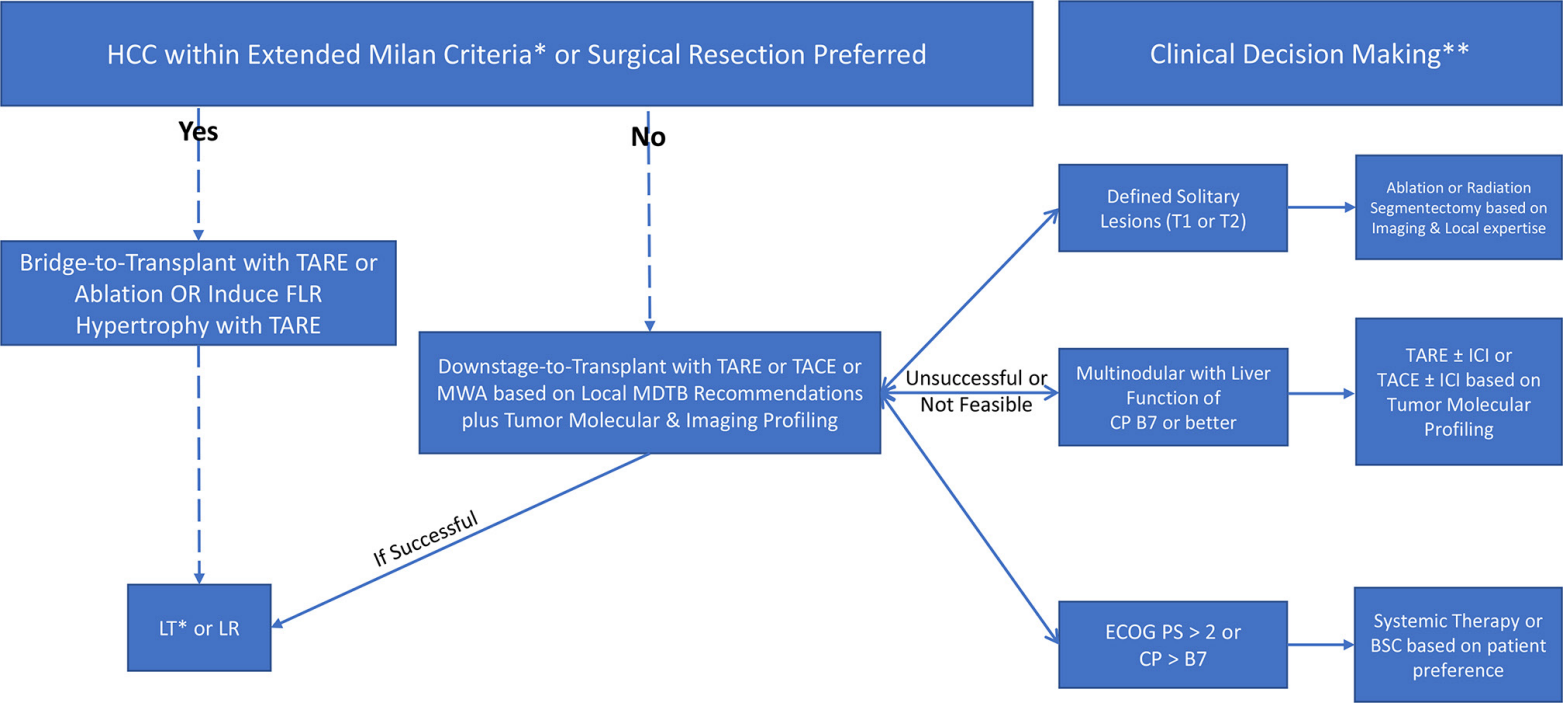
Simplified proposed algorithm of future management of hepatocellular carcinoma (HCC). *Assuming MELD-Na exception points granted as well as other local transplant requirements met **Combination of local expertise, multidisciplinary tumor board and patient preference. Abbreviations: TARE = transarterial radioembolization LT = liver transplant LR = liver resection TACE = transarterial chemoembolization CP = Child Pugh ICI = immune checkpoint inhibitors BSC = best supportive care PS = performance score MDTB = multidisciplinary tumor board

Three major organizations provide clinical practice recommendations for the management of HCC – the American Association for the Study of Liver Disease (AASLD), European Association for the Study of the Liver (EASL), and the Asian Pacific Association for the Study of the Liver (APASL).^109^ Newer iterations of the AASLD, EASL, and APASL guidelines that incorporate the BCLC update are pending.^10,49,110^ In the interim, Figure 2 depicts a proposed future HCC treatment algorithm for patients either awaiting or patients ineligible for liver transplant. The key argument behind this proposed rationale is that radioembolization can be considered alongside and in tandem with surgical and ablative modalities. As medicine continues to extend the lifespan of patients suffering from HCC, let us remember that prevention is the best cure, particularly in today’s times of isolation and strained healthcare access. Therefore, the aforementioned discussion should not supplant investments in public health measures combating obesity, improving mental health, and increasing vaccination uptake.

## References

[1] . Sung H, Ferlay J, Siegel RL, Laversanne M, Soerjomataram I, Jemal A, et al . Global Cancer Statistics 2020: GLOBOCAN Estimates of Incidence and Mortality Worldwide for 36 Cancers in 185 Countries. CA: A Cancer Journal for Clinicians. 2021;71(3):209-49. 33538338 10.3322/caac.21660

[2] . Saito A, Toyoda H, Kobayashi M, Koiwa Y, Fujii H, Fujita K, et al . Prediction of early recurrence of hepatocellular carcinoma after resection using digital pathology images assessed by machine learning. Modern Pathology. 2021;34(2):417-25. 32948835 10.1038/s41379-020-00671-z PMC7817520

[3] Llovet JM, De Baere T, Kulik L, Haber PK, Greten TF, Meyer T, et al . Locoregional therapies in the era of molecular and immune treatments for hepatocellular carcinoma. Nat Rev Gastroenterol Hepatol 2021; 18: 293–313. doi: 10.1038/s41575-020-00395-0 33510460

[4] . Reig M, Forner A, Rimola J, Ferrer-Fábrega J, Burrel M, Garcia-Criado A, et al . BCLC strategy for prognosis prediction and treatment recommendation Barcelona Clinic Liver Cancer (BCLC) staging system. The 2022 update. J Hepatol. 2021. 10.1016/j.jhep.2021.11.018 PMC8866082 34801630

[5] . Park J-W, Chen M, Colombo M, Roberts LR, Schwartz M, Chen P-J, et al . Global patterns of hepatocellular carcinoma management from diagnosis to death: the BRIDGE Study. Liver International. 2015;35(9):2155-66. 25752327 10.1111/liv.12818 PMC4691343

[6] Lucatelli P, Guiu B . 2022 update of BCLC treatment algorithm of HCC: what’s new for interventional radiologists? Cardiovasc Intervent Radiol 2022; 45: 275–76. doi: 10.1007/s00270-021-03047-1 35088139

[7] Llovet JM, Lencioni R . MRECIST for HCC: performance and novel refinements. J Hepatol 2020; 72: 288–306: S0168-8278(19)30592-6. doi: 10.1016/j.jhep.2019.09.026 31954493 PMC12452114

[8] Alem Z, Murray TE, Egri C, Chung J, Liu D, Elsayes KM, et al . Treatment response assessment following transarterial radioembolization for hepatocellular carcinoma. Abdom Radiol (NY) 2021; 46: 3596–3614. doi: 10.1007/s00261-021-03095-8 33909092

[9] Lingiah VA, Niazi M, Olivo R, Paterno F, Guarrera JV, Pyrsopoulos NT . Liver transplantation beyond milan criteria. J Clin Transl Hepatol 2020; 8: 69–75. doi: 10.14218/JCTH.2019.00050 32274347 PMC7132012

[10] Marrero JA, Kulik LM, Sirlin CB, Zhu AX, Finn RS, Abecassis MM, et al . Diagnosis, staging, and management of hepatocellular carcinoma: 2018 practice guidance by the american association for the study of liver diseases. Hepatology 2018; 68: 723–50. doi: 10.1002/hep.29913 29624699

[11] Mazzaferro V, Regalia E, Doci R, Andreola S, Pulvirenti A, Bozzetti F, et al . Liver transplantation for the treatment of small hepatocellular carcinomas in patients with cirrhosis. N Engl J Med 1996; 334: 693–99. doi: 10.1056/NEJM199603143341104 8594428

[12] . Warhadpande SLACKJTMPI. Pocketbook of Clinical IR : a concise guide to interventional radiology2019.

[13] Xu X, Lau WY, Yang T . (n.d.). The updated BCLC staging system needs further refinement: A surgeon’s perspective. J Hepatol. 10.1016/j.jhep.2022.01.002 35066088

[14] Crocetti L, Bozzi E, Scalise P, Bargellini I, Lorenzoni G, Ghinolfi D, et al . Locoregional treatments for bridging and downstaging HCC to liver transplantation. Cancers (Basel) 2021; 13(21): 5558. doi: 10.3390/cancers13215558 34771720 PMC8583584

[15] Gabr A, Kulik L, Mouli S, Riaz A, Ali R, Desai K, et al . Liver transplantation following yttrium-90 radioembolization: 15-year experience in 207-patient cohort. Hepatology 2021; 73: 998–1010. doi: 10.1002/hep.31318 32416631

[16] Abulkhir A, Limongelli P, Healey AJ, Damrah O, Tait P, Jackson J, et al . Preoperative portal vein embolization for major liver resection: a meta-analysis. Ann Surg 2008; 247: 49–57. doi: 10.1097/SLA.0b013e31815f6e5b 18156923

[17] Bekki Y, Marti J, Toshima T, Lewis S, Kamath A, Argiriadi P, et al . A comparative study of portal vein embolization versus radiation lobectomy with yttrium-90 micropheres in preparation for liver resection for initially unresectable hepatocellular carcinoma. Surgery 2021; 169: 1044–51: S0039-6060(20)30847-3. doi: 10.1016/j.surg.2020.12.012 33648768

[18] Guiu B, Garin E, Allimant C, Edeline J, Salem R . TARE in hepatocellular carcinoma: from the right to the left of BCLC. Cardiovasc Intervent Radiol 2022. doi: 10.1007/s00270-022-03072-8 35149884

[19] Finn RS, Qin S, Ikeda M, Galle PR, Ducreux M, Kim T-Y, et al . Atezolizumab plus bevacizumab in unresectable hepatocellular carcinoma. N Engl J Med 2020; 382: 1894–1905. doi: 10.1056/NEJMoa1915745 32402160

[20] Abou-Alfa GK, Chan SL, Kudo M, Lau G, Kelley RK, Furuse J, et al . Phase 3 randomized, open-label, multicenter study of tremelimumab (T) and durvalumab (D) as first-line therapy in patients (pts) with unresectable hepatocellular carcinoma (uhcc): HIMALAYA. JCO 2022; 40: 379. doi: 10.1200/JCO.2022.40.4_suppl.379

[21] Llovet JM, Castet F, Heikenwalder M, Maini MK, Mazzaferro V, Pinato DJ, et al . Immunotherapies for hepatocellular carcinoma. Nat Rev Clin Oncol 2022; 19: 151–72. doi: 10.1038/s41571-021-00573-2 34764464

[22] Delis SG, Dervenis C . Selection criteria for liver resection in patients with hepatocellular carcinoma and chronic liver disease. World J Gastroenterol 2008; 14: 3452–60. doi: 10.3748/wjg.14.3452 18567070 PMC2716604

[23] Ahmed M, Solbiati L, Brace CL, Breen DJ, Callstrom MR, Charboneau JW, et al . Image-guided tumor ablation: standardization of terminology and reporting criteria--a 10-year update. Radiology 2014; 273: 241–60. doi: 10.1148/radiol.14132958 24927329 PMC4263618

[24] Habibollahi P, Sheth RA, Cressman ENK . Histological correlation for radiofrequency and microwave ablation in the local control of hepatocellular carcinoma (HCC) before liver transplantation: A comprehensive review. Cancers (Basel) 2020; 13(1): E104. doi: 10.3390/cancers13010104 PMC7795634 33396289

[25] Majumdar A, Roccarina D, Thorburn D, Davidson BR, Tsochatzis E, Gurusamy KS . Management of people with early- or very early-stage hepatocellular carcinoma: an attempted network meta-analysis. Cochrane Database Syst Rev 2017; 3: CD011650. doi: 10.1002/14651858.CD011650.pub2 28351116 PMC6464490

[26] Jia JB, Zhang D, Ludwig JM, Kim HS . Radiofrequency ablation versus resection for hepatocellular carcinoma in patients with child-pugh A liver cirrhosis: A meta-analysis. Clin Radiol 2017; 72: 1066–75: S0009-9260(17)30410-5. doi: 10.1016/j.crad.2017.07.024 28851491

[27] Nault J-C, Sutter O, Nahon P, Ganne-Carrié N, Séror O . Percutaneous treatment of hepatocellular carcinoma: state of the art and innovations. J Hepatol 2018; 68: 783–97: S0168-8278(17)32351-6. doi: 10.1016/j.jhep.2017.10.004 29031662

[28] Vietti Violi N, Duran R, Guiu B, Cercueil J-P, Aubé C, Digklia A, et al . Efficacy of microwave ablation versus radiofrequency ablation for the treatment of hepatocellular carcinoma in patients with chronic liver disease: a randomised controlled phase 2 trial. Lancet Gastroenterol Hepatol 2018; 3: 317–25: S2468-1253(18)30029-3. doi: 10.1016/S2468-1253(18)30029-3 29503247

[29] Chong CCN, Lee KF, Cheung SYS, Chu CCM, Fong AKW, Wong J, et al . Prospective double-blinded randomized controlled trial of microwave versus radiofrequency ablation for hepatocellular carcinoma (mcrfa trial). HPB (Oxford) 2020; 22: 1121–27: S1365-182X(20)30023-X. doi: 10.1016/j.hpb.2020.01.008 32044268

[30] Radosevic A, Quesada R, Serlavos C, Sánchez J, Zugazaga A, Sierra A, et al . Microwave versus radiofrequency ablation for the treatment of liver malignancies: a randomized controlled phase 2 trial. Sci Rep 2022; 12(1): 316. doi: 10.1038/s41598-021-03802-x 35013377 PMC8748896

[31] Vouche M, Habib A, Ward TJ, Kim E, Kulik L, Ganger D, et al . Unresectable solitary hepatocellular carcinoma not amenable to radiofrequency ablation: multicenter radiology-pathology correlation and survival of radiation segmentectomy. Hepatology 2014; 60: 192–201. doi: 10.1002/hep.27057 24691943

[32] Pommergaard H-C, Rostved AA, Adam R, Thygesen LC, Salizzoni M, Gómez Bravo MA, et al . Locoregional treatments before liver transplantation for hepatocellular carcinoma: a study from the european liver transplant registry. Transpl Int 2018; 31: 531–39. doi: 10.1111/tri.13123 29380442

[33] Ogawa K, Takada Y . Role of pretransplant treatments for patients with hepatocellular carcinoma waiting for liver transplantation. Cancers 2019; 14: 396. doi: 10.3390/cancers14020396 PMC8773674 35053558

[34] Ahmed M, Kumar G, Moussa M, Wang Y, Rozenblum N, Galun E, et al . Hepatic radiofrequency ablation-induced stimulation of distant tumor growth is suppressed by c-met inhibition. Radiology 2016; 279: 103–17. doi: 10.1148/radiol.2015150080 26418615 PMC4819900

[35] Shi L, Wang J, Ding N, Zhang Y, Zhu Y, Dong S, et al . Inflammation induced by incomplete radiofrequency ablation accelerates tumor progression and hinders PD-1 immunotherapy. Nat Commun 2019; 10(1. doi: 10.1038/s41467-019-13204-3 PMC6883042 31780645

[36] Greten TF, Mauda-Havakuk M, Heinrich B, Korangy F, Wood BJ . Combined locoregional-immunotherapy for liver cancer. J Hepatol 2019; 70: 999–1007: S0168-8278(19)30074-1. doi: 10.1016/j.jhep.2019.01.027 30738077 PMC6462230

[37] O’Leary C, Mahler M, Soulen MC . Curative-intent therapies in localized hepatocellular carcinoma. Curr Treat Options Oncol 2020; 21(4): 31. doi: 10.1007/s11864-020-0725-3 32193784

[38] Malagari K, Pomoni M, Kelekis A, Pomoni A, Dourakis S, Spyridopoulos T, et al . Prospective randomized comparison of chemoembolization with doxorubicin-eluting beads and bland embolization with beadblock for hepatocellular carcinoma. Cardiovasc Intervent Radiol 2010; 33: 541–51. doi: 10.1007/s00270-009-9750-0 19937027

[39] Brown KT, Do RK, Gonen M, Covey AM, Getrajdman GI, Sofocleous CT, et al . Randomized trial of hepatic artery embolization for hepatocellular carcinoma using doxorubicin-eluting microspheres compared with embolization with microspheres alone. J Clin Oncol 2016; 34: 2046–53. doi: 10.1200/JCO.2015.64.0821 26834067 PMC4966514

[40] Boulin M, Guiu B . Chemoembolization or bland embolization for hepatocellular carcinoma: the question is still unanswered. J Clin Oncol 2017; 35: 256–57. doi: 10.1200/JCO.2016.67.2915 28056196

[41] Rand T, Loewe C, Schoder M, Schmook MT, Peck-Radosavljevic M, Kettenbach J, et al . Arterial embolization of unresectable hepatocellular carcinoma with use of microspheres, lipiodol, and cyanoacrylate. Cardiovasc Intervent Radiol 2005; 28: 313–18. doi: 10.1007/s00270-004-0153-y 15886943

[42] Kouri BE . Interventional oncology: optimizing transarterial therapies for the treatment of hepatic malignancy. Techniques in Vascular and Interventional Radiology 2005; 21: 205–22. doi: 10.1053/j.tvir.2018.07.002 30545499

[43] Lo C-M, Ngan H, Tso W-K, Liu C-L, Lam C-M, Poon RT-P, et al . Randomized controlled trial of transarterial lipiodol chemoembolization for unresectable hepatocellular carcinoma. Hepatology 2002; 35: 1164–71. doi: 10.1053/jhep.2002.33156 11981766

[44] Llovet JM, Real MI, Montaña X, Planas R, Coll S, Aponte J, et al . Arterial embolisation or chemoembolisation versus symptomatic treatment in patients with unresectable hepatocellular carcinoma: a randomised controlled trial. The Lancet 2005; 359: 1734–39. doi: 10.1016/S0140-6736(02)08649-X 12049862

[45] Gaba RC, Lokken RP, Hickey RM, Lipnik AJ, Lewandowski RJ, Salem R, et al . Quality improvement guidelines for transarterial chemoembolization and embolization of hepatic malignancy. Journal of Vascular and Interventional Radiology 2005; 28: 1210–1223. doi: 10.1016/j.jvir.2017.04.025 28669744

[46] de Baere T, Arai Y, Lencioni R, Geschwind J-F, Rilling W, Salem R, et al . Treatment of liver tumors with lipiodol TACE: technical recommendations from experts opinion. Cardiovasc Intervent Radiol 2016; 39: 334–43. doi: 10.1007/s00270-015-1208-y 26390875

[47] Salem R, Lewandowski RJ . Chemoembolization and radioembolization for hepatocellular carcinoma. Clin Gastroenterol Hepatol 2013; 11: 604–11; S1542-3565(13)00097-9. doi: 10.1016/j.cgh.2012.12.039 23357493 PMC3800021

[48] Varela M, Real MI, Burrel M, Forner A, Sala M, Brunet M, et al . Chemoembolization of hepatocellular carcinoma with drug eluting beads: efficacy and doxorubicin pharmacokinetics. J Hepatol 2007; 46: 474–81. doi: 10.1016/j.jhep.2006.10.020 17239480

[49] European Association for the Study of the Liver . EASL clinical practice guidelines: management of hepatocellular carcinoma. J Hepatol 2018; 69: 182–236: S0168-8278(18)30215-0. doi: 10.1016/j.jhep.2018.03.019 29628281

[50] Lencioni R, Llovet JM, Han G, Tak WY, Yang J, Guglielmi A, et al . Sorafenib or placebo plus TACE with doxorubicin-eluting beads for intermediate stage HCC: the SPACE trial. J Hepatol 2016; 64: 1090–98: S0168-8278(16)00018-0. doi: 10.1016/j.jhep.2016.01.012 26809111

[51] . Kudo M, Ueshima K, Ikeda M, Torimura T, Tanabe N, Aikata H, et al . Randomised, multicentre prospective trial of transarterial chemoembolisation (TACE) plus sorafenib as compared with TACE alone in patients with hepatocellular carcinoma: TACTICS trial. Gut. 2020;69(8):1492-501. 31801872 10.1136/gutjnl-2019-318934 PMC7398460

[52] Duran R, Chapiro J, Schernthaner RE, Geschwind J-FH . Systematic review of catheter-based intra-arterial therapies in hepatocellular carcinoma: state of the art and future directions. BJR 2015; 88: 20140564. doi: 10.1259/bjr.20140564 25978585 PMC4651391

[53] . Kaseb AO, Vence L, Blando J, Yadav SS, Ikoma N, Pestana RC, et al . Immunologic Correlates of Pathologic Complete Response to Preoperative Immunotherapy in Hepatocellular Carcinoma. Cancer Immunol Res. 2019;7(9):1390-5. 31289040 10.1158/2326-6066.CIR-18-0605 PMC7726707

[54] . Cerrito L, Annicchiarico BE, Iezzi R, Gasbarrini A, Pompili M, Ponziani FR. Treatment of hepatocellular carcinoma in patients with portal vein tumor thrombosis: Beyond the known frontiers. World J Gastroenterol. 2019;25(31):4360-82. 31496618 10.3748/wjg.v25.i31.4360 PMC6710186

[55] Yang B, Liang J, Qu Z, Yang F, Liao Z, Gou H . Transarterial strategies for the treatment of unresectable hepatocellular carcinoma: A systematic review. PLoS One 2020; 15(2): e0227475. doi: 10.1371/journal.pone.0227475 32074102 PMC7029952

[56] Dhondt E, Lambert B, Hermie L, Huyck L, Vanlangenhove P, Geerts A, et al . Yttrium-90 radioembolization versus drug-eluting beads chemoembolization for unresectable hepatocellular carcinoma: results from the TRACE phase 2 randomized controlled trial. Journal of Hepatology 2022; 77: S107: 211806. 10.1016/S0168-8278(22)00602-X 35258371

[57] Dhondt E, Hermie L, Verhelst X, Lambert B, Defreyne L . 4:21 PM abstract no. 307 transarterial radioembolization versus drug-eluting beads chemoembolization for treatment of inoperable early and intermediate hepatocellular carcinoma: interim results of the randomized controlled TRACE trial. Journal of Vascular and Interventional Radiology 2020; 31: S140. doi: 10.1016/j.jvir.2019.12.360

[58] Rahman SI-U, Nunez-Herrero L, Berkes JL . Position 2: transarterial radioembolization should be the primary locoregional therapy for unresectable hepatocellular carcinoma. Clin Liver Dis (Hoboken) 2020; 15: 74–76. doi: 10.1002/cld.908 32226620 PMC7098664

[59] Skef W, Agarwal M, Mikolajczyk AE . Position 1: transarterial chemoembolization should be the primary locoregional therapy for unrespectable hepatocelluar carcinoma. Clin Liver Dis (Hoboken) 2020; 15: 71–73. doi: 10.1002/cld.909 32226619 PMC7098666

[60] Kwon JH, Kim GM, Han K, Won JY, Kim MD, Lee DY, et al . Safety and efficacy of transarterial radioembolization combined with chemoembolization for bilobar hepatocellular carcinoma: A single-center retrospective study. Cardiovasc Intervent Radiol 2018; 41: 459–65. doi: 10.1007/s00270-017-1826-7 29067511

[61] Dezarn WA, Cessna JT, DeWerd LA, Feng W, Gates VL, Halama J, et al . Recommendations of the american association of physicists in medicine on dosimetry, imaging, and quality assurance procedures for 90Y microsphere brachytherapy in the treatment of hepatic malignancies. Med Phys 2011; 38: 4824–45. doi: 10.1118/1.3608909 21928655

[62] Kao Y-H, Steinberg JD, Tay Y-S, Lim GK, Yan J, Townsend DW, et al . Post-radioembolization yttrium-90 PET/CT - part 1: diagnostic reporting. EJNMMI Res 2013; 3: 56. doi: 10.1186/2191-219X-3-56 23883566 PMC3726297

[63] Kao Y-H, Steinberg JD, Tay Y-S, Lim GK, Yan J, Townsend DW, et al . Post-radioembolization yttrium-90 PET/CT - part 2: dose-response and tumor predictive dosimetry for resin microspheres. EJNMMI Res 2013; 3: 57. doi: 10.1186/2191-219X-3-57 23885971 PMC3733999

[64] Salem R, Thurston KG . Radioembolization with 90yttrium microspheres: A state-of-the-art brachytherapy treatment for primary and secondary liver malignancies. part 1: technical and methodologic considerations. J Vasc Interv Radiol 2006; 17: 1251–78. doi: 10.1097/01.RVI.0000233785.75257.9A 16923973

[65] Miller FH, Lopes Vendrami C, Gabr A, Horowitz JM, Kelahan LC, Riaz A, et al . Evolution of radioembolization in treatment of hepatocellular carcinoma: A pictorial review. Radiographics 2021; 41: 1802–18. doi: 10.1148/rg.2021210014 34559587

[66] . Lewandowski RJ, Gabr A, Abouchaleh N, Ali R, Al Asadi A, Mora RA, et al . Radiation Segmentectomy: Potential Curative Therapy for Early Hepatocellular Carcinoma. Radiology. 2018;287(3):1050-8. 29688155 10.1148/radiol.2018171768

[67] Kim PH, Choi SH, Kim JH, Park SH . Comparison of radioembolization and sorafenib for the treatment of hepatocellular carcinoma with portal vein tumor thrombosis: A systematic review and meta-analysis of safety and efficacy. Korean J Radiol 2019; 20: 385. doi: 10.3348/kjr.2018.0496 30799569 PMC6389804

[68] Kulik LM, Carr BI, Mulcahy MF, Lewandowski RJ, Atassi B, Ryu RK, et al . Safety and efficacy of 90Y radiotherapy for hepatocellular carcinoma with and without portal vein thrombosis. Hepatology 2019; 47: 71–81. doi: 10.1002/hep.21980 18027884

[69] Ahn JC, Lauzon M, Luu M, Friedman ML, Kosari K, Nissen N, et al . Transarterial radioembolization versus systemic treatment for hepatocellular carcinoma with macrovascular invasion: analysis of the u.s. national cancer database. J Nucl Med 2019; 62: 1692–1701. doi: 10.2967/jnumed.121.261954 PMC8612185 33837067

[70] Salem R, Gordon AC, Mouli S, Hickey R, Kallini J, Gabr A, et al . Y90 radioembolization significantly prolongs time to progression compared with chemoembolization in patients with hepatocellular carcinoma. Gastroenterology 2019; 151: 1155–1163. doi: 10.1053/j.gastro.2016.08.029 PMC5124387 27575820

[71] Kirchner T, Marquardt S, Werncke T, Kirstein MM, Brunkhorst T, Wacker F, et al . Comparison of health-related quality of life after transarterial chemoembolization and transarterial radioembolization in patients with unresectable hepatocellular carcinoma. Abdom Radiol (NY) 2019; 44: 1554–61. doi: 10.1007/s00261-018-1802-y 30311050

[72] Salem R, Gilbertsen M, Butt Z, Memon K, Vouche M, Hickey R, et al . Increased quality of life among hepatocellular carcinoma patients treated with radioembolization, compared with chemoembolization. Clinical Gastroenterology and Hepatology 2013; 11: 1358–1365. doi: 10.1016/j.cgh.2013.04.028 23644386

[73] Kolligs FT, Bilbao JI, Jakobs T, Iñarrairaegui M, Nagel JM, Rodriguez M, et al . Pilot randomized trial of selective internal radiation therapy vs. chemoembolization in unresectable hepatocellular carcinoma. Liver Int 2015; 35: 1715–21. doi: 10.1111/liv.12750 25443863

[74] Lemieux S, Buies A, F Turgeon A, Hallet J, Daigle G, Côté F, et al . Effect of yttrium-90 transarterial radioembolization in patients with non-surgical hepatocellular carcinoma: A systematic review and meta-analysis. PLoS One 2021; 16(3): e0247958. doi: 10.1371/journal.pone.0247958 33662011 PMC7932100

[75] Salem R, Johnson GE, Kim E, Riaz A, Bishay V, Boucher E, et al . Yttrium-90 radioembolization for the treatment of solitary, unresectable HCC: the LEGACY study. Hepatology 2021; 74: 2342–52. doi: 10.1002/hep.31819 33739462 PMC8596669

[76] Iñarrairaegui M, Sangro B . Selective internal radiation therapy approval for early HCC: what comes next? Hepatology 2021; 74: 2333–35. doi: 10.1002/hep.32054 34245592

[77] Matsumoto MM, Mouli S, Saxena P, Gabr A, Riaz A, Kulik L, et al . Comparing real world, personalized, multidisciplinary tumor board recommendations with BCLC algorithm: 321-patient analysis. Cardiovasc Intervent Radiol 2021; 44: 1070–80. doi: 10.1007/s00270-021-02810-8 33825060

[78] Garin E, Tselikas L, Guiu B, Chalaye J, Edeline J, de Baere T, et al . Personalised versus standard dosimetry approach of selective internal radiation therapy in patients with locally advanced hepatocellular carcinoma (DOSISPHERE-01): a randomised, multicentre, open-label phase 2 trial. Lancet Gastroenterol Hepatol 2021; 6: 17–29: S2468-1253(20)30290-9. doi: 10.1016/S2468-1253(20)30290-9 33166497

[79] Lewandowski RJ, Salem R . Radioembolisation with personalised dosimetry: improving outcomes for patients with advanced hepatocellular carcinoma. Lancet Gastroenterol Hepatol 2021; 6: 2–3: S2468-1253(20)30306-X. doi: 10.1016/S2468-1253(20)30306-X 33166498

[80] Salem R, Padia SA, Lam M, Bell J, Chiesa C, Fowers K, et al . Clinical and dosimetric considerations for Y90: recommendations from an international multidisciplinary working group. Eur J Nucl Med Mol Imaging 2019; 46: 1695–1704. doi: 10.1007/s00259-019-04340-5 31098749

[81] Mahvash A, Chartier S, Turco M, Habib P, Griffith S, Brown S, et al . A prospective, multicenter, open-label, single-arm clinical trial design to evaluate the safety and efficacy of ^90^Y resin microspheres for the treatment of unresectable HCC: the doorway90 (duration of objective response with arterial ytrrium-90) study. BMC Gastroenterol 2022; 22(1): 151. doi: 10.1186/s12876-022-02204-1 35346070 PMC8962126

[82] Lam M, Garin E, Maccauro M, Kappadath SC, Sze DY, Turkmen C, et al . A global evaluation of advanced dosimetry in transarterial radioembolization of hepatocellular carcinoma with yttrium-90: the TARGET study. Eur J Nucl Med Mol Imaging 2022. doi: 10.1007/s00259-022-05774-0 PMC9308596 35394152

[83] Teyateeti A, Mahvash A, Long JP, Abdelsalam ME, Avritscher R, Chasen B, et al . Survival outcomes for yttrium-90 transarterial radioembolization with and without sorafenib for unresectable hepatocellular carcinoma patients. J Hepatocell Carcinoma 2020; 7: 117–31. doi: 10.2147/JHC.S248314 32984089 PMC7500841

[84] Kim KJ, Kim JH, Lee SJ, Lee EJ, Shin EC, Seong J . Radiation improves antitumor effect of immune checkpoint inhibitor in murine hepatocellular carcinoma model. Oncotarget 2017; 8: 41242–55. doi: 10.18632/oncotarget.17168 28465485 PMC5522235

[85] Tai D, Loke K, Gogna A, Kaya NA, Tan SH, Hennedige T, et al . Radioembolisation with Y90-resin microspheres followed by nivolumab for advanced hepatocellular carcinoma (CA 209-678): a single arm, single centre, phase 2 trial. Lancet Gastroenterol Hepatol 2021; 6: 1025–35: S2468-1253(21)00305-8. doi: 10.1016/S2468-1253(21)00305-8 34695377

[86] Yau T, Park JW, Finn RS, Cheng AL, Mathurin P, Edeline J, et al . (n.d.). LBA38_PR - checkmate 459: A randomized, multi-center phase III study of nivolumab (NIVO) vs sorafenib (SOR) as first-line (1L) treatment in patients (pts) with advanced hepatocellular carcinoma (ahcc). Annals of Oncology 2019;30:V874-V5.

[87] Swersky A, Kulik L, Kalyan A, Grace K, Caicedo JC, Lewandowski RJ, et al . Contemporary algorithm for the management of hepatocellular carcinoma in 2021: the northwestern approach. Semin Intervent Radiol 2021; 38: 432–37. doi: 10.1055/s-0041-1735528 34629710 PMC8497077

[88] Doemel LA, Santana JG, Savic LJ, Gaupp FML, Borde T, Petukhova-Greenstein A, et al . Comparison of metabolic and immunologic responses to transarterial chemoembolization with different chemoembolic regimens in a rabbit VX2 liver tumor model. Eur Radiol 2022; 32: 2437–47. doi: 10.1007/s00330-021-08337-3 34718844 PMC9359419

[89] Boulin M, Schmitt A, Delhom E, Cercueil J-P, Wendremaire M, Imbs D-C, et al . Improved stability of lipiodol-drug emulsion for transarterial chemoembolisation of hepatocellular carcinoma results in improved pharmacokinetic profile: proof of concept using idarubicin. Eur Radiol 2016; 26: 601–9. doi: 10.1007/s00330-015-3855-4 26060065

[90] Chai NX, Chapiro J . Therapy of intermediate-stage hepatocellular carcinoma: current evidence and clinical practice. Semin Intervent Radiol 2020; 37: 456–65. doi: 10.1055/s-0040-1719186 33328701 PMC7732559

[91] Chu HH, Kim JH, Yoon H-K, Ko H-K, Gwon DI, Kim PN, et al . Chemoembolization combined with radiofrequency ablation for medium-sized hepatocellular carcinoma: A propensity-score analysis. J Vasc Interv Radiol 2019; 30: 1533–43: S1051-0443(19)30551-2. doi: 10.1016/j.jvir.2019.06.006 31471190

[92] Gui CH, Baey S, D’cruz RT, Shelat VG . Trans-arterial chemoembolization + radiofrequency ablation versus surgical resection in hepatocellular carcinoma - A meta-analysis. Eur J Surg Oncol 2020; 46: 763–71: S0748-7983(20)30004-4. doi: 10.1016/j.ejso.2020.01.004 31937433

[93] Li HZ, Tan J, Tang T, An TZ, Li JX, Xiao YD . Chemoembolization plus microwave ablation vs chemoembolization alone in unresectable hepatocellular carcinoma beyond the milan criteria: A propensity scoring matching study. J Hepatocell Carcinoma 2021; 8: 1311–22. doi: 10.2147/JHC.S338456 34754838 PMC8570378

[94] Levillain H, Bagni O, Deroose CM, Dieudonné A, Gnesin S, Grosser OS, et al . International recommendations for personalised selective internal radiation therapy of primary and metastatic liver diseases with yttrium-90 resin microspheres. Eur J Nucl Med Mol Imaging 2021; 48: 1570–84. doi: 10.1007/s00259-020-05163-5 33433699 PMC8113219

[95] Plachouris D, Tzolas I, Gatos I, Papadimitroulas P, Spyridonidis T, Apostolopoulos D, et al . A deep-learning-based prediction model for the biodistribution of ^90^ y microspheres in liver radioembolization. Med Phys 2021; 48: 7427–38. doi: 10.1002/mp.15270 34628667

[96] Gabr A, Ranganathan S, Mouli SK, Riaz A, Gates VL, Kulik L, et al . Streamlining radioembolization in UNOS T1/T2 hepatocellular carcinoma by eliminating lung shunt estimation. Journal of Hepatology 2020; 72: 1151–58. doi: 10.1016/j.jhep.2020.02.024 32145255

[97] Garin E, Palard X, Rolland Y . Personalised dosimetry in radioembolisation for HCC: impact on clinical outcome and on trial design. Cancers 2020; 12: 1557. 10.3390/cancers12061557 32545572 PMC7353030

[98] . Martins F, Sofiya L, Sykiotis GP, Lamine F, Maillard M, Fraga M, et al . Adverse effects of immune-checkpoint inhibitors: epidemiology, management and surveillance. Nature Reviews Clinical Oncology. 2019;16(9):563-80. 10.1038/s41571-019-0218-0 31092901

[99] Wei W, Rosenkrans ZT, Liu J, Huang G, Luo Q-Y, Cai W . ImmunoPET: concept, design, and applications. Chem Rev 2020; 120: 3787–3851. doi: 10.1021/acs.chemrev.9b00738 32202104 PMC7265988

[100] Gregory J, Dioguardi Burgio M, Corrias G, Vilgrain V, Ronot M . Evaluation of liver tumour response by imaging. JHEP Reports 2020; 2: 100100. doi: 10.1016/j.jhepr.2020.100100 32514496 PMC7267412

[101] McGlynn KA, Petrick JL, El‐Serag HB . Epidemiology of hepatocellular carcinoma. Hepatology 2021; 73: 4–13. doi: 10.1002/hep.31288 PMC7577946 32319693

[102] . Pfister D, Núñez NG, Pinyol R, Govaere O, Pinter M, Szydlowska M, et al . NASH limits anti-tumour surveillance in immunotherapy-treated HCC. Nature. 2021;592(7854):450-6. 33762733 10.1038/s41586-021-03362-0 PMC8046670

[103] Anstee QM, Reeves HL, Kotsiliti E, Govaere O, Heikenwalder M . From NASH to HCC: current concepts and future challenges. Nat Rev Gastroenterol Hepatol 2021; 16: 411–28. doi: 10.1038/s41575-019-0145-7 31028350

[104] Caruso S, O’Brien DR, Cleary SP, Roberts LR, Zucman‐Rossi J . Genetics of hepatocellular carcinoma: approaches to explore molecular diversity. Hepatology 2021; 73: 14–26. doi: 10.1002/hep.31394 32463918

[105] Craig AJ, von Felden J, Garcia-Lezana T, Sarcognato S, Villanueva A . Tumour evolution in hepatocellular carcinoma. Nat Rev Gastroenterol Hepatol 2021; 17: 139–52. doi: 10.1038/s41575-019-0229-4 31792430

[106] Rebouissou S, Nault JC . Advances in molecular classification and precision oncology in hepatocellular carcinoma. J Hepatol 2020; 72: 215–29: S0168-8278(19)30484-2. doi: 10.1016/j.jhep.2019.08.017 31954487

[107] Liu JKH, Irvine AF, Jones RL, Samson A . Immunotherapies for hepatocellular carcinoma. Cancer Med 2022; 11: 571–91. doi: 10.1002/cam4.4468 34953051 PMC8817091

[108] Xiao Y, Chen J, Zhou H, Zeng X, Ruan Z, Pu Z, et al . Combining p53 mrna nanotherapy with immune checkpoint blockade reprograms the immune microenvironment for effective cancer therapy. Nat Commun 2022; 13(1): 758. doi: 10.1038/s41467-022-28279-8 35140208 PMC8828745

[109] Kim T-H, Kim SY, Tang A, Lee JM . Comparison of international guidelines for noninvasive diagnosis of hepatocellular carcinoma: 2018 update. Clin Mol Hepatol 2019; 25: 245–63. doi: 10.3350/cmh.2018.0090 30759967 PMC6759428

[110] Omata M, Cheng A-L, Kokudo N, Kudo M, Lee JM, Jia J, et al . Asia-pacific clinical practice guidelines on the management of hepatocellular carcinoma: a 2017 update. Hepatol Int 2017; 11: 317–70. doi: 10.1007/s12072-017-9799-9 28620797 PMC5491694

